# Short-term swallowing outcomes associated with inductive electrical stimulation combined with tongue pressure resistance feedback training in post-stroke dysphagia

**DOI:** 10.3389/fmed.2026.1911750

**Published:** 2026-09-01

**Authors:** Fangfang Zhang, Min Lu, Qiong Yi

**Affiliations:** Department of Rehabilitation Medicine, The Affiliated Huai'an No.1 People's Hospital of Nanjing Medical University, Huai'an, Jiangsu, China

**Keywords:** biofeedback, dysphagia, inductive electrical stimulation, rehabilitation, stroke, tongue pressure resistance training

## Abstract

**Objective:**

To investigate the short-term swallowing outcomes associated with videofluoroscopy-guided inductive electrical stimulation combined with tongue pressure resistance feedback training in patients with post-stroke dysphagia.

**Methods:**

This non-randomized, single-center prospective study enrolled 238 patients with post-stroke dysphagia between January 2024 and December 2025. Patients were allocated according to preference to receive conventional swallowing rehabilitation or a a time-matched integrated program in which inductive electrical stimulation and tongue pressure resistance feedback training were incorporated within the same 30-minute rehabilitation session. The primary outcome was the change in Penetration-Aspiration Scale (PAS) score from baseline to post-treatment. Secondary outcomes included Functional Oral Intake Scale (FOIS), Swallowing Quality of Life questionnaire, Standardized Swallowing Assessment, videofluoroscopic kinematic parameters, surface electromyography parameters, maximum isometric tongue pressure, and post-treatment swallowing status improvement rate. Adjusted ANCOVA or logistic regression models were used to estimate treatment-associated differences.

**Results:**

Several baseline imbalances were observed, including stroke type, modified Rankin Scale score, baseline FOIS, baseline SWAL-QOL, and baseline tongue pressure. After adjustment for baseline values and clinically relevant covariates, the intervention group showed a greater reduction in PAS score than the control group, with an adjusted difference of −0.21 points; 95% CI, −0.37–−0.05; *P* = 0.012. The intervention group also showed greater improvements in FOIS, SWAL-QOL, SSA, tongue pressure, thyroid cartilage and hyoid bone displacement, and swallowing time. The post-treatment swallowing status improvement rate was 89.9% in the intervention group and 73.1% in the control group, corresponding to an absolute rate difference of 16.8%, 95% CI, 7.2%–26.4%. In adjusted logistic regression, the intervention was associated with higher odds of clinical response, with an adjusted OR of 1.71, 95% CI, 1.15–2.59. Ordinal logistic sensitivity analyses for post-treatment PAS and FOIS yielded consistent findings. No serious adverse events were reported.

**Conclusion:**

Videofluoroscopy-guided inductive electrical stimulation combined with tongue pressure resistance feedback training was associated with greater short-term improvements in swallowing-related clinical, kinematic, and tongue pressure outcomes compared with conventional rehabilitation. Given the preference-based allocation and the multicomponent nature of the intervention, these findings should be interpreted as associative rather than definitive evidence of causal association.

## Introduction

Stroke remains the second leading cause of mortality and the third leading cause of disability worldwide, carrying a profound global burden (1, 2). Dysphagia, a common and debilitating complication of stroke, has an incidence ranging from 51% to 73% (3). Post-stroke dysphagia (PSD) is primarily caused by lesions in the cerebral cortex, brainstem, or cranial nerves, leading to uncoordinated neuromuscular control during the oral, pharyngeal, and esophageal phases of swallowing (4). Clinically, PSD manifests as delayed swallowing initiation, nasal regurgitation, repetitive swallowing efforts, choking, and a diminished cough reflex. These deficits significantly predispose patients to severe complications, including aspiration pneumonia, malnutrition, and dehydration, which independently prolong hospital stays and increase the risk of mortality and long-term disability (5). Therefore, optimizing rehabilitation strategies for PSD remains clinically important.

Current therapeutic approaches for PSD are diverse, including oral sensory-motor training, traditional peripheral neuromuscular electrical stimulation (NMES), non-invasive brain stimulation such as transcranial magnetic stimulation, direct swallowing maneuvers, biofeedback therapies, and balloon dilatation (6, 7). However, traditional rehabilitation modalities often yield suboptimal outcomes. Standard electrical stimulation primarily targets superficial muscles, leaving severe tongue or pharyngeal weakness unaddressed (8). Similarly, conventional balloon-based tongue devices suffer from instability in positioning and imprecise feedback. In contrast, inductive electrical stimulation allows mobile stimulation of both extraoral and selected intraoral sites and may provide additional sensory-motor input (9, 10). Alongside this, modern multi-point tongue pressure biofeedback provides localized, real-time assessments. Recent studies have suggested potential benefits in rebuilding tongue strength and reducing aspiration (11, 12). Ultimately, the clinical heterogeneity of PSD demands precise evaluation. Videofluoroscopy (VFS) is widely regarded as a reference standard for this purpose, providing real-time, quantifiable visualization of swallowing biomechanics to guide these advanced interventions (13).

While existing literature extensively covers isolated rehabilitation modalities for PSD, comprehensive interventions that integrate precise VFS evaluation with both inductive electrical stimulation and targeted tongue pressure resistance training remain underexplored. Therefore, this non-randomized prospective study was designed to evaluate short-term clinical and physiological swallowing outcomes associated with a VFS-guided, time-matched multimodal rehabilitation program integrating inductive electrical stimulation and tongue pressure resistance feedback training. We hypothesize that this integrated approach, tailored by VFS findings, will be associated with greater short-term clinical outcomes and objective swallowing kinematics compared to conventional rehabilitation in patients with PSD.

## Methods

### Study design and participants

This is a non-randomized, single-center, prospective cohort study conducted at the Department of Rehabilitation Medicine, The Affiliated Huai'an No. 1 People's Hospital of Nanjing Medical University, between January 2024 and December 2025. The study protocol was initially approved by the ethical committee on April 14, 2021 (JS-2021-001-01) and underwent annually review (KY-2026-153-01). Written informed consent was obtained from all patients or their legal guardians before enrollment. This study was not a registered trial. The inclusion criteria were as follows: (1) met the diagnostic criteria for stroke confirmed by CT or MRI, accompanied by dysphagia confirmed by VFS; (2) first-ever stroke with a disease duration of ≤ 3 months; (3) conscious, with stable vital signs, and capable of cooperating with rehabilitation assessments and training; (4) Functional Oral Intake Scale (FOIS) score ≤ 6; (5) aged > 18 years.

The exclusion criteria included: (1) presence of malignant tumors; (2) severe psychological disorders, cognitive impairment, or epilepsy; (3) absolute contraindications for electrical stimulation therapy (e.g., acute purulent inflammation, implanted cardiac pacemakers); (4) allergy to contrast agents, precluding VFS assessment; (5) pre-existing severe respiratory diseases (e.g., chronic obstructive pulmonary disease, asthma, lung cancer); (6) severe dysfunction of the heart, liver, or kidneys; (7) inability to comply with the treatment protocol or refused participation.

**Figure 1:**
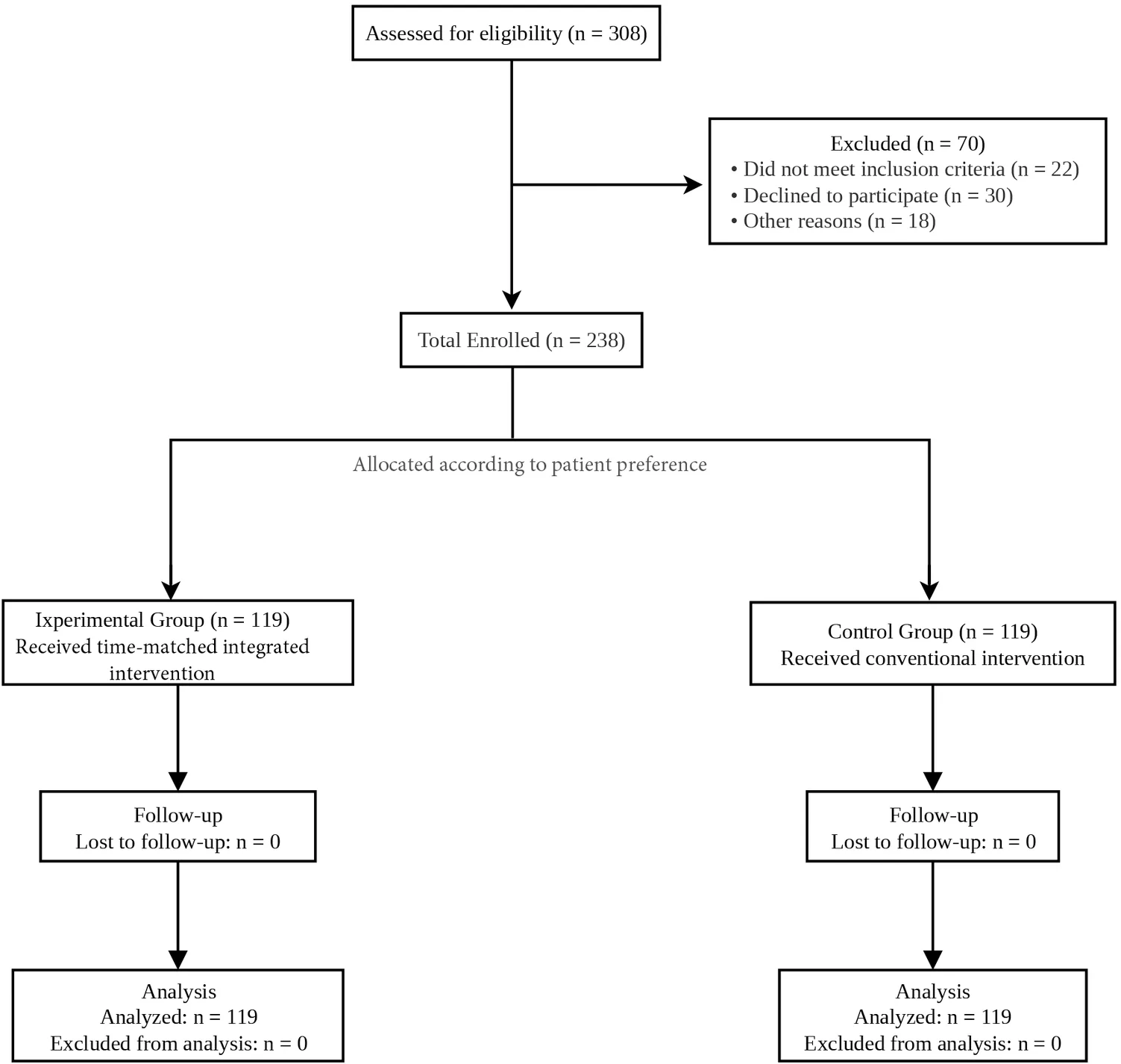
Participant flow diagram. A total of 308 patients with post-stroke dysphagia were assessed for eligibility. Seventy patients were excluded before enrollment, including 22 who did not meet the inclusion criteria, 30 who declined to participate, and 18 who were excluded for other reasons. Finally, 238 patients were enrolled and allocated according to patient preference to the control group (*n* = 119) or the intervention group (*n* = 119). All enrolled patients completed the 3-week follow-up and were included in the final analysis. No patients were lost to follow-up or excluded from analysis.

A participant flow diagram was constructed to improve the transparency of patient enrollment and follow-up (Figure 1). The diagram summarizes the numbers of patients assessed for eligibility, excluded before enrollment, allocated to the control or intervention group according to patient preference, completing the 3-week follow-up, and included in the final analysis. After being informed of the available rehabilitation options, patients or their legal guardians selected the treatment program according to preference. No random allocation or matching procedure was used. A blinded physician assessed the outcomes after treatment program.

### Interventions

Both groups received therapist-supervised rehabilitation for the same total duration: 30 min per session, once daily, 5 days per week, for 3 consecutive weeks. Prior to study initiation, therapists underwent standardized training on device operation and the treatment protocol.

Patients in the control group received conventional swallowing rehabilitation, including oral sensory-motor training, external NMES, swallowing maneuvers, and VFS-guided direct feeding training. During direct feeding training, food texture, bolus volume, and compensatory postures were individualized according to VFS findings. Patients in the intervention group received a time-matched integrated rehabilitation program. Within the same 30-minute session, inductive electrical stimulation and tongue pressure resistance feedback training were incorporated into the conventional rehabilitation procedures rather than added as extra treatment time. Inductive electrical stimulation was delivered for approximately 20 min using a handheld mobile probe to stimulate the suprahyoid region and selected intraoral sites according to VFS-identified swallowing deficits. Inductive electrical stimulation was delivered using a low-frequency swallowing neuromuscular stimulator in handheld mobile mode. The stimulation parameters were set as follows: pulse width, 100 μs; frequency, 80 Hz; stimulation duration, 3–5 s; and interval, 5–10 s. A handheld probe was used to stimulate the suprahyoid muscle group externally and selected intraoral sites, including the tongue, soft palate, palatopharyngeal arch, uvula, and posterior pharyngeal wall. The stimulation sites were selected according to baseline VFS findings and the patient's predominant swallowing deficits, such as impaired tongue base retraction, delayed pharyngeal triggering, reduced hyolaryngeal excursion, or pharyngeal residue. Current intensity was gradually titrated to the patient's maximum tolerable motor threshold without pain. A square auxiliary electrode was placed on the posterior neck. Inductive electrical stimulation was performed for approximately 20 min within the 30-minute rehabilitation session and was combined with swallowing-related exercises when appropriate. Tongue pressure resistance feedback training was performed using a digital tongue pressure biofeedback device. Maximum isometric tongue pressure was first measured, and the training target was set at 60%–80% of the patient's maximum value. Under visual feedback, patients were instructed to place the pressure probe on the midline of the tongue and press it firmly against the hard palate to reach and maintain the target pressure. Ten repetitions constituted one set, with short rest intervals between repetitions to prevent fatigue. This training was incorporated into the same 30-minute intervention session. Therefore, the total daily supervised treatment duration was identical between groups. A component-level summary of treatment content and duration is provided in Supplementary Table 1 to clarify treatment fidelity and the time-matched nature of the two rehabilitation programs.

### Outcome measures

Assessments were conducted at baseline (before treatment) and after the 3-week intervention period. Post-treatment swallowing status was descriptively assessed using the Kubota Water Swallowing Test. A score of Grade 1–2 indicated “Functional Recovery”; Grade 3 indicated “Improved”; and Grade >3 indicated “Unchanged ”. Clinical improvement rate = [(Functional Recovery + Improved)/Total cases]  ×  100%. Because this classification was based on post-treatment status rather than change from baseline, it was analyzed as a descriptive secondary endpoint and was not used as the primary outcome. Functional Oral Intake Scale (FOIS) is a 7-point observer-rated scale evaluating the patient's diet level. Level 1 indicates nothing by mouth, while Level 7 indicates a totally unrestricted oral diet. Higher scores represent better swallowing function (14). Standardized Swallowing Assessment (SSA) is a comprehensive clinical evaluation of dysphagia severity. The total score is 46 points, with higher scores indicating more severe swallowing impairment (15). Penetration-Aspiration Scale (PAS) was evaluated during VFS, the PAS is an 8-point scale defining the depth of airway invasion and whether the material is expelled. A score of 1 indicates no airway entry, while a score of 8 indicates silent aspiration (material enters below the vocal folds with no effort to clear). Higher scores indicate an increased risk of aspiration (16). Swallowing Quality of Life (SWAL-QOL) is a validated patient-reported outcome measure specifically designed for dysphagia. Higher scores indicate a better quality of life and reduced dysphagia-related psychological and social burden (17). Kinematic Analysis (VFS Parameters) was performed using digital frame-by-frame analysis of the VFS recordings by specialized software. The maximum anterior and superior displacement (in millimeters) of the Thyroid Cartilage (TC) and Hyoid Bone (HB) during a 5 mL thick liquid swallow were calculated. Greater displacement values signify superior hyolaryngeal excursion capacity (18). Surface electromyography (sEMG) signal was recorded using VitalStim® Plus Electrotherapy and sEMG System (Model 5923-3; Chattanooga, Enovis, UK). After skin cleaning with alcohol, surface electrodes were placed over the submental region along the anterior belly of the suprahyoid muscle complex, with an inter-electrode distance of approximately 2 cm. Signals were sampled at 250 Hz and filtered using a 18–370 Hz band-pass filter. Patients performed 5 swallows of the standardized bolus, and swallowing onset and offset were identified from the sEMG waveform by a blinded assessor according to a standardized protocol. The maximum raw peak amplitude and swallowing duration were extracted for analysis. Because maximum voluntary isometric contraction was not recorded, amplitude normalization was not performed. Electrode placement and skin preparation procedures were kept as consistent as possible across assessments. Therefore, sEMG amplitude was interpreted as an exploratory indicator of submental muscle activity rather than a direct measure of muscle strength, coordination, or neuromuscular recruitment (19). Maximum Isometric Tongue Pressure (MITP): The MITP was assessed using the digital tongue pressure biofeedback device (Model LGC-2352DS, Longest, China). Patients were instructed to compress the air-filled balloon probe against their hard palate as forcefully as possible. The highest value from three consecutive trials, separated by a 1-minute rest interval, was recorded in kilopascals (kPa). This measurement was performed at baseline and post-treatment to evaluate changes in tongue muscle strength.

The primary outcome was the change in PAS score from baseline to post-treatment. Because the study was not prospectively registered, the designation of PAS change as the primary outcome should be interpreted as an analysis-defined primary endpoint based on its clinical relevance and VFS-based objectivity. Secondary outcomes included changes in FOIS, SWAL-QOL, SSA, maximum isometric tongue pressure, VFS-derived kinematic parameters, sEMG parameters, and post-treatment swallowing status improvement rate. Adverse events were extracted from electronic medical records system according to routine clinical and rehabilitation records. Because a prespecified standardized adverse-event checklist was not used, safety findings were analyzed descriptively.

Because of the nature of the rehabilitation interventions, blinding of patients and treating therapists was not feasible. However, all outcome assessments, including VFS interpretation, sEMG data extraction, and clinical scale scoring, were performed by independent rehabilitation physicians who were blinded to treatment allocation and were not involved in treatment delivery.

### VFS swallowing study and kinematic analysis

VFS swallowing study was performed with patients seated in an upright lateral position. A 5-mL bolus of thickened liquid mixed with barium sulfate suspension was used for standardized assessment. The barium concentration was 50% weight/volume, and the liquid consistency corresponded to International Dysphagia Diet Standardisation Initiative Level 2. Fluoroscopic images were recorded at a frame rate of 30 frames per second and stored digitally for offline frame-by-frame analysis.

For kinematic measurements, a radiopaque calibration marker of known diameter was placed at the level of the neck during VFS recording to correct for image magnification. The coordinate system was established using cervical vertebral landmarks. The line connecting the anterior-inferior borders of the second and fourth cervical vertebrae was defined as the vertical reference axis, and a perpendicular line was used as the horizontal reference axis. The hyoid bone was tracked using the anterior-superior margin of the hyoid body, and the thyroid cartilage was tracked using the anterior-superior margin of the thyroid cartilage. The maximum superior and anterior displacement of the hyoid bone and thyroid cartilage from the resting position to the point of maximal excursion during swallowing was calculated in millimeters. The VFSS was performed by a multidisciplinary team including a senior speech-language pathologist and a radiologist. Five bolus consistencies were prepared using barium sulfate and a thickening agent, categorized according to the International Dysphagia Diet Standardisation Initiative or equivalent scales: Level 0 (thin liquid), Level 1 (slightly thick), Level 2 (mildly thick), Level 3 (moderately thick), and Level 4 (solid food). Patients were positioned in a seated, upright posture with the head in a neutral position, facing forward. Following a standardized protocol, the consistencies were administered in the specific order of Level 2, 1, 0, 3, and 4 under x-ray fluoroscopy. Swallowing sequences were recorded and subsequently analyzed using specialized digital image acquisition and analysis software to determine temporal parameters (e.g., oral and pharyngeal transit times). All procedures strictly adhered to radiation protection principles, minimizing fluoroscopy exposure time while ensuring high-quality image capture. For each patient, the trial representing their best performance was selected for final analysis.

VFS recordings were reviewed by experienced rehabilitation physicians who were blinded to treatment allocation and were not involved in treatment delivery. PAS grading and kinematic measurements were performed according to a standardized protocol.

### Statistical analysis

Continuous variables were summarized as mean ± standard deviation or median with interquartile range, as appropriate. Categorical variables were summarized as frequencies and percentages. Baseline differences between groups were assessed using independent-sample t tests, Mann–Whitney U tests, chi-square tests, or Fisher's exact tests, as appropriate. Standardized mean differences (SMDs) were additionally calculated to assess baseline balance between groups, with an absolute SMD < 0.10 considered indicative of good balance.

The primary outcome was the change in PAS score from baseline to post-treatment. The primary analysis was performed using an ANCOVA model, with post-treatment PAS score as the dependent variable, treatment group as the main independent variable, and baseline PAS score, stroke type, NIHSS score, modified Rankin Scale score, and disease duration as covariates. Covariates were selected based on their established clinical prognostic value in stroke recovery and observed baseline imbalances (absolute SMD > 0.10). Additionally, the baseline value of each respective outcome measure was included to account for pre-treatment severity. Therefore, the adjusted estimate represents the between-group difference in post-treatment PAS score conditional on baseline PAS and covariates, which was used to assess treatment-associated differences in change over time.

For secondary continuous outcomes, treatment-associated differences were estimated using ANCOVA models adjusted for the corresponding baseline value of each outcome and the same clinically relevant covariates. Adjusted mean differences with 95% confidence intervals were reported. For the post-treatment Kubota-based favorable swallowing status, logistic regression was used as an exploratory analysis to estimate odds ratios and 95% confidence intervals. Because FOIS and PAS are ordinal variables, proportional odds logistic regression models were performed as sensitivity analyses.

To further assess robustness, a propensity score-adjusted sensitivity analysis was performed. The propensity score was estimated using a multivariable logistic regression model including clinically relevant baseline variables and variables with an absolute SMD >0.10. The estimated propensity score was then entered as an additional covariate into the adjusted regression models for the primary and key secondary outcomes.

No formal *a priori* sample-size calculation was performed. The sample size was based on the number of eligible consecutive patients enrolled during the study period. Therefore, the analyses should be considered exploratory. A two-sided *P* value < 0.05 was considered statistically significant.

## Results

### Participant flow

A total of 308 patients were assessed for eligibility during the study period. Of these, 70 patients were excluded: 22 did not meet the inclusion criteria, 30 declined to participate, and 18 were excluded for other reasons (including incomplete baseline VFS assessment (n = 10), transfer to another department or hospital before treatment initiation (*n* = 3), inability to complete the planned rehabilitation schedule (*n* = 2), and incomplete baseline clinical data (*n* = 3)). Finally, 238 patients were enrolled and allocated according to patient preference, including 119 patients in the control group and 119 patients in the intervention group. Enrollment was closed after 119 patients in each group completing the treatment program and follow-up. No random allocation or statistical matching procedure was used. All patients completed the 15 planned therapist-supervised sessions, and no missed, interrupted, or discontinued sessions were recorded in either group (Supplementary Table 2). Therefore, complete-case analysis was performed without imputation. The total supervised treatment duration was 30 min per session in both groups, and no patients were lost to follow-up or excluded from the final analysis. The detailed participant flow is shown in Figure 1.

### Baseline characteristics

The baseline demographic, stroke-related, and dysphagia-related characteristics of the 238 analyzed patients are summarized in Table 1. Several baseline imbalances were observed between the two groups. Stroke type differed significantly between groups, with a higher proportion of hemorrhagic stroke in the control group and a higher proportion of ischemic stroke in the intervention group. The intervention group also had higher modified Rankin Scale scores, lower baseline FOIS scores, lower baseline SWAL-QOL scores, and substantially lower baseline tongue pressure. Therefore, subsequent outcome analyses were adjusted for baseline outcome values and clinically relevant covariates.

**Table 1:** Baseline demographic, stroke-related, and dysphagia-related characteristics of the study participants.

Variables	Intervention group (*n* = 119)	Control group (*n* = 119)	*P* value	SMD
Age, years	64.80 (12.41)	64.57 (11.23)	0.880	0.020
Male sex, *n* (%)	84 (70.6)	80 (67.2)	0.674	0.073
Disease duration, days	17.04 (2.91)	17.29 (3.13)	0.534	0.081
Stroke type, *n* (%)			0.027	0.342
Ischemic stroke	70 (58.8)	52 (43.7)		
Hemorrhagic stroke	49 (41.2)	67 (56.3)		
NIHSS score	15.19 (4.89)	14.82 (3.07)	0.485	0.091
modified Rankin Scale	3.51 (0.55)	3.23 (0.42)	<0.001	0.583
Lesion location, *n* (%)			0.254	0.231
Brainstem lesion	20 (16.8)	11 (9.2)		
Supratentorial lesion	61 (51.3)	65 (54.6)		
Bilateral lesion	4 (3.4)	6 (5.0)		
Others	34 (28.5)	37 (31.2)		
Baseline FOIS	2.08 (0.67)	2.24 (0.61)	0.043	0.263
Baseline PAS	5.38 (1.10)	5.39 (1.10)	0.906	0.015
Baseline SSA	28.75 (3.31)	29.14 (3.52)	0.374	0.116
Baseline SWAL-QOL	85.87 (9.82)	90.01 (8.89)	0.001	0.441
Baseline tongue pressure, kPa	15.93 (2.89)	19.59 (2.41)	<0.001	1.373
Nasogastric tube feeding, *n* (%)	119 (100.0)	119 (100.0)	-	0.000
Pneumonia before intervention, *n* (%)	82 (68.9)	75 (63.0)	0.412	0.124
Tracheostomy, *n* (%)	19 (16.0)	17 (14.3)	0.856	0.047
Hypertension, *n* (%)	91 (76.5)	90 (75.6)	>0.999	0.02
Diabetes mellitus, *n* (%)	34 (28.6)	42 (35.3)	0.330	0.145
Coronary heart disease, *n* (%)	14 (11.8)	13 (10.9)	>0.999	0.026
Atrial fibrillation, *n* (%)	14 (11.8)	13 (10.9)	>0.999	0.026

Continuous variables are presented as mean (standard deviation), and categorical variables are presented as number and percentage. Between-group comparisons were performed using Welch's two-sample t test or the Mann–Whitney U test for continuous or ordinal variables, as appropriate, and Pearson's chi-square test or Fisher's exact test for categorical variables, as appropriate. Stroke type was analyzed as a single mutually exclusive categorical variable rather than as separate binary variables. Standardized mean differences (SMDs) were calculated to assess baseline balance between groups; an absolute SMD < 0.10 was considered indicative of good balance. A two-sided *P* value < 0.05 was considered statistically significant. NIHSS, National Institutes of Health Stroke Scale; mRS, modified rankin scale; FOIS, functional oral intake scale; PAS, penetration-aspiration scale; SSA, standardized swallowing assessment; SWAL-QOL, swallowing quality of life; SMD, standardized mean difference; SD, standard deviation.

### Post-treatment Kubota-based swallowing status

Post-treatment Kubota-based swallowing status are presented in Table 2. After 3 weeks of treatment, the proportion of patients with favorable post-treatment Kubota-based swallowing status was higher in the intervention group. In the intervention group, 107 of 119 patients were classified as clinical improvement, corresponding to a response rate of 89.9%. In the control group, 87 of 119 patients were classified as clinical improvement, corresponding to a response rate of 73.1%. The absolute rate difference was 16.8%; 95% CI, 7.2%–26.4%. The crude odds ratio for clinical response was 3.28; 95% CI, 1.59–6.75. After adjustment for stroke type, NIHSS score, modified Rankin Scale score, disease duration, baseline PAS score, and baseline FOIS score, the intervention remained associated with higher odds of clinical response, with an adjusted odds ratio of 1.71; 95% CI, 1.15–2.59.

**Table 2:** Post-treatment kubota-based swallowing status between the two groups (*N* = 238).

Group	Clinical improvement, *n* (%)	Unchanged, *n* (%)	Rate Difference (95% CI)	Unadjusted OR (95% CI)	Adjusted OR^a^ (95% CI)	Adjusted *P*-value
Control (*n* = 119)	87 (73.1%)	32 (26.9%)	-	-	-	-
Intervention (*n* = 119)	107 (89.9%)	12 (10.1%)	16.8% (7.2%–26.4%)	3.28 (1.59–6.75)^b^	1.71 (1.15–2.59)^c^	0.009

a Adjusted for stroke type, NIHSS, mRS, disease duration, baseline PAS, and baseline FOIS score using logistic regression.

b Calculated from crude 2 × 2 contingency table.

c Adjusted ORs were estimated using multivariable logistic regression with the control group as the reference.

### Primary outcome: PAS score

**Figure 2:**
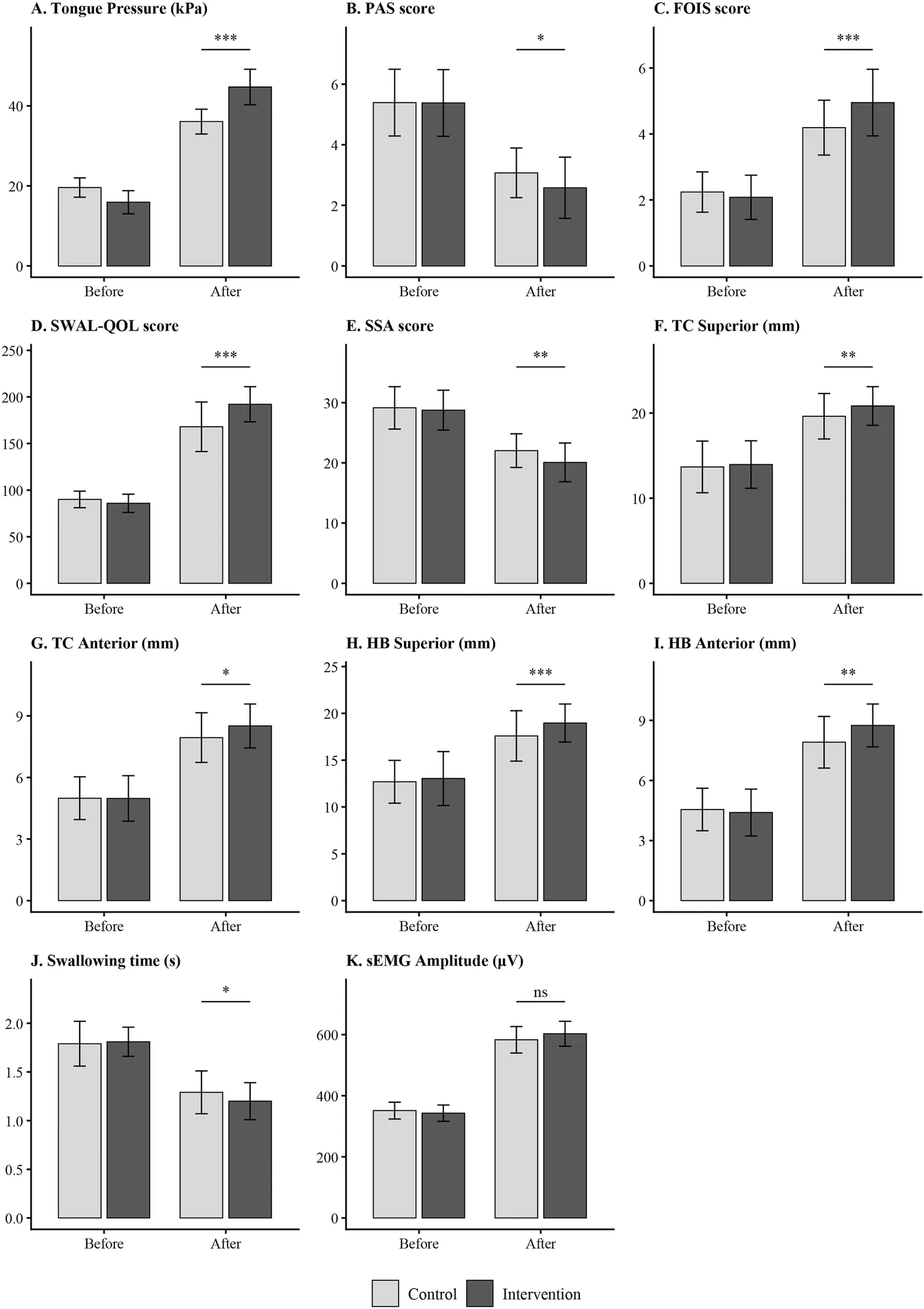
Comparison of swallowing outcomes. Comparison of clinical and kinematic swallowing outcomes between the control and intervention groups. Data are presented as mean ± standard deviation (SD). **(A)** Tongue pressure (kPa); **(B)** Penetration-Aspiration Scale (PAS) score; **(C)** Functional Oral Intake Scale (FOIS) score; **(D)** Swallowing Quality of Life (SWAL-QOL) score; **(E)** Standardized Swallowing Assessment (SSA) score; **(F,G)** Superior and anterior displacement of the thyroid cartilage (TC); **(H,I)** Superior and anterior displacement of the hyoid bone (HB); **(J)** Total swallowing time; **(K)** Surface electromyography (sEMG) amplitude of the suprahyoid muscle group. Light grey bars represent the Control group, and dark grey bars represent the intervention group. Statistical significance for the inter-group comparison at the “After” time point (adjusted for baseline via ANCOVA) is indicated as follows: ***P < 0.001, **P < 0.01, *P < 0.05, and ns, non-significant (*P* = 0.051).

The primary outcome was the change in PAS score from baseline to post-treatment. As shown in Table 3 and Figure 2B, PAS scores decreased in both groups after the 3-week treatment period. The control group showed a mean reduction from 5.39 ± 1.10–3.07 ± 0.82, whereas the intervention group showed a mean reduction from 5.38 ± 1.10–2.58 ± 1.01. The mean change was −2.33 ± 1.43 in the control group and −2.80 ± 1.46 in the intervention group. In the ANCOVA model adjusted for baseline PAS score, stroke type, NIHSS score, modified Rankin Scale score, and disease duration, the intervention group showed a greater reduction in PAS score, with an adjusted difference of −0.21 points; 95% CI, −0.37–−0.05; *P* = 0.012.

**Table 3:** Outcomes of intervention on primary and secondary swallowing outcomes (*N* = 238).

Outcome Measures	Group	Baseline (Mean ± SD)	Post-treatment (Mean ± SD)	Change (Mean ± SD)	Adjusted treatment-associated difference (95% CI)^a^	*P*-value
Primary Outcomes						
PAS score	Control	5.39 ± 1.10	3.07 ± 0.82	−2.33 ± 1.43	—	—
	Intervention	5.38 ± 1.10	2.58 ± 1.01	−2.80 ± 1.46	−0.21 (−0.37, −0.05)	0.012
Secondary Outcomes						
FOIS score	Control	2.24 ± 0.61	4.19 ± 0.83	1.95 ± 0.70	—	—
	Intervention	2.08 ± 0.67	4.95 ± 1.01	2.87 ± 1.08	0.35 (0.19, 0.52)	< 0.001
SWAL-QOL	Control	90.01 ± 8.89	167.96 ± 26.58	77.95 ± 24.21	—	—
	Intervention	85.87 ± 9.82	192.15 ± 18.88	106.28 ± 20.54	14.30 (9.49, 19.20)	< 0.001
SSA score	Control	29.14 ± 3.52	22.03 ± 2.80	−7.11 ± 4.40	—	—
	Intervention	28.75 ± 3.31	20.08 ± 3.21	−8.67 ± 4.73	−0.70 (−1.18, −0.21)	0.005
Tongue Pressure (kPa)	Control	19.59 ± 2.41	36.07 ± 3.11	16.48 ± 3.11	—	—
	Intervention	15.93 ± 2.89	44.71 ± 4.44	28.77 ± 3.42	5.65 (5.06, 6.25)	< 0.001
sEMG Amplitude (μV)	Control	351.40 ± 27.43	583.10 ± 43.33	231.70 ± 52.75	—	—
	Intervention	342.97 ± 26.58	602.63 ± 40.75	259.66 ± 53.06	10.70 (−0.05, 21.40)	0.051
Kinematic Parameters						
TC Superior (mm)	Control	13.67 ± 3.04	19.63 ± 2.67	5.96 ± 4.39	—	—
	Intervention	13.96 ± 2.79	20.84 ± 2.27	6.88 ± 3.69	0.60 (0.15, 1.05)	0.009
TC Anterior (mm)	Control	4.99 ± 1.04	7.94 ± 1.21	2.95 ± 1.63	—	—
	Intervention	4.98 ± 1.11	8.51 ± 1.07	3.53 ± 1.39	0.25 (0.01, 0.48)	0.040
HB Superior (mm)	Control	12.69 ± 2.29	17.59 ± 2.69	4.91 ± 3.43	—	—
	Intervention	13.04 ± 2.88	18.97 ± 2.03	5.93 ± 3.59	0.60 (0.27, 0.94)	< 0.001
HB Anterior (mm)	Control	4.55 ± 1.06	7.91 ± 1.29	3.36 ± 1.45	—	—
	Intervention	4.40 ± 1.17	8.75 ± 1.07	4.35 ± 1.60	0.33 (0.13, 0.53)	0.001
Swallowing time (s)	Control	1.79 ± 0.23	1.29 ± 0.22	−0.50 ± 0.31	—	—
	Intervention	1.81 ± 0.15	1.20 ± 0.19	−0.61 ± 0.27	−0.04 (−0.07, −0.01)	0.013

Data are presented as mean ± standard deviation (SD) for raw scores.

Clinical Interpretation: For FOIS, SWAL-QOL, Tongue Pressure, sEMG amplitude, and all displacement parameters (TC/HB), a positive adjusted difference indicates a greater improvement in the intervention group compared to the control group. For PAS score, SSA score, and Swallowing time, a negative adjusted difference indicates a superior reduction in symptoms or duration in the intervention group. CI, confidence interval; FOIS, functional oral intake scale; HB, hyoid bone; PAS, penetration-aspiration scale; sEMG, surface electromyography; SSA, standardized swallowing assessment; SWAL-QOL, swallowing quality of life questionnaire; TC, thyroid cartilage.

a Adjusted differences represent between-group differences in post-treatment values estimated from ANCOVA models adjusted for the corresponding baseline outcome value and covariates.

### Secondary clinical swallowing outcomes

Secondary swallowing-related clinical outcomes are summarized in Table 3 and Figures 2C–E. FOIS scores increased in both groups, with a larger improvement in the intervention group. The mean FOIS change was 1.95 ± 0.70 in the control group and 2.87 ± 1.08 in the intervention group. After adjustment, the intervention was associated with a higher post-treatment FOIS score, with an adjusted difference of 0.35; 95% CI, 0.19–0.52; *P* < 0.001.

SWAL-QOL scores also increased in both groups. The mean change was 77.95 ± 24.21 in the control group and 106.28 ± 20.54 in the intervention group. The adjusted between-group difference was 14.30; 95% CI, 9.49–19.20; *P* < 0.001, favoring the intervention group.

SSA scores decreased after treatment in both groups. The mean reduction was −7.11 ± 4.40 in the control group and −8.67 ± 4.73 in the intervention group. After adjustment, the intervention group showed a greater reduction in SSA score, with an adjusted difference of −0.70; 95% CI, −1.18–−0.21; *P* = 0.005.

### Tongue pressure

Maximum isometric tongue pressure outcomes are presented in Table 3 and Figure 2A. Baseline tongue pressure was lower in the intervention group than in the control group, indicating an important baseline imbalance. After treatment, tongue pressure increased in both groups. The control group increased from 19.59 ± 2.41 kPa to 36.07 ± 3.11 kPa, whereas the intervention group increased from 15.93 ± 2.89 kPa to 44.71 ± 4.44 kPa. The mean change was 16.48 ± 3.11 kPa in the control group and 28.77 ± 3.42 kPa in the intervention group. After ANCOVA adjustment, the intervention was associated with a greater increase in tongue pressure, with an adjusted difference of 5.65 kPa; 95% CI, 5.06–6.25; *P* < 0.001.

### VFS kinematic parameters

VFS-derived kinematic outcomes are shown in Table 3 and Figures 2F–I. Superior and anterior displacement of both the thyroid cartilage and hyoid bone increased after treatment in both groups. After adjustment, the intervention group showed greater improvement in thyroid cartilage superior displacement, with an adjusted difference of 0.60 mm; 95% CI, 0.15–1.05; *P* = 0.009. Thyroid cartilage anterior displacement was also greater in the intervention group, with an adjusted difference of 0.25 mm; 95% CI, 0.01–0.48; *P* = 0.040.

**Figure 3:**
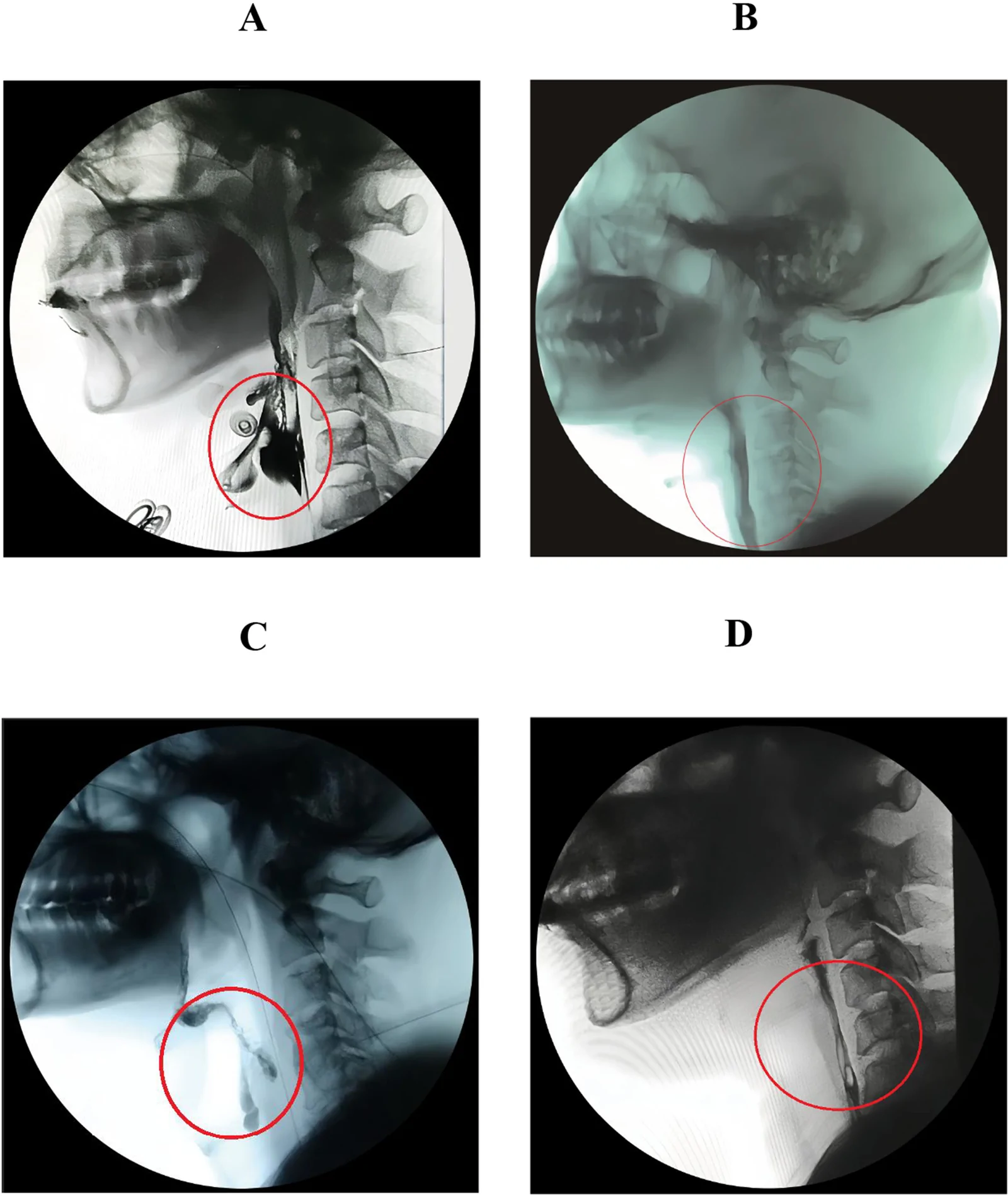
Representative VFS images before and after the intervention. Representative VFS images from two patients in the intervention group are shown for illustrative purposes. **(A)** Pre-treatment image of Patient 1 during intake of 5 mL Level 2 liquid, showing reduced tongue base retraction, pharyngeal residue, insufficient hyolaryngeal excursion, and penetration. **(B)** Post-treatment image of Patient 1 showing reduced pharyngeal residue and no visible penetration or aspiration during the selected swallow. **(C)** Pre-treatment image of Patient 2 showing impaired swallowing safety and efficiency with residue and airway invasion. **(D)** Post-treatment image of Patient 2 showing improved bolus clearance and no visible penetration or aspiration during the selected swallow. These representative images are illustrative and should not be interpreted as evidence of complete normalization of swallowing function.

Similarly, hyoid bone superior displacement improved more in the intervention group, with an adjusted difference of 0.60 mm; 95% CI, 0.27–0.94; *P* < 0.001. Hyoid bone anterior displacement also favored the intervention group, with an adjusted difference of 0.33 mm; 95% CI, 0.13–0.53; *P* = 0.001. Representative VFS images before and after treatment are shown in Figure 3.

### sEMG parameters and swallowing time

Surface electromyography and swallowing time outcomes are presented in Table 3 and Figures 2J,K. Swallowing time decreased in both groups, from 1.79 ± 0.23 s to 1.29 ± 0.22 s in the control group and from 1.81 ± 0.15 s to 1.20 ± 0.19 s in the intervention group. After adjustment, the intervention group showed a greater reduction in swallowing time, with an adjusted difference of −0.04 s; 95% CI, −0.07–−0.01; *P* = 0.013.

Maximum sEMG amplitude increased in both groups. The control group increased from 351.40 ± 27.43 μV to 583.10 ± 43.33 μV, and the intervention group increased from 342.97 ± 26.58 μV to 602.63 ± 40.75 μV. However, after adjustment, the between-group difference did not reach conventional statistical significance, with an adjusted difference of 10.70 μV; 95% CI, −0.05–21.40; *P* = 0.051.

### Sensitivity analysis for ordinal outcomes

Because PAS and FOIS are ordinal scales, proportional odds logistic regression models were performed as sensitivity analyses. As shown in Table 4, the intervention group had lower odds of being in a worse post-treatment PAS category, with an adjusted OR of 0.59; 95% CI, 0.45–0.77; *P* < 0.001. For post-treatment FOIS, the intervention group had higher odds of achieving a better functional oral intake level, with an adjusted OR of 2.45; 95% CI, 1.84–3.31; *P* < 0.001. These ordinal sensitivity analyses were consistent with the primary adjusted analyses.

**Table 4:** Sensitivity analysis: proportional odds logistic regression for post-treatment FOIS and PAS levels.

Outcome Measure	Independent Variable	Adjusted OR (95% CI)	z value	*P* value
PAS Grade	Group (Intervention vs. Control)	0.59 (0.45–0.77)	−3.89	< 0.001
	Baseline PAS	0.93 (0.75–1.14)	−0.72	0.469
	Stroke type	2.06 (0.63–6.76)	1.30	0.194
	NIHSS	1.03 (0.94–1.13)	0.59	0.553
	mRS	1.01 (0.45–2.27)	0.02	0.984
	Duration	1.00 (0.92–1.08)	−0.09	0.929
FOIS Grade	Group (Intervention vs. Control)	2.45 (1.84–3.31)	6.04	< 0.001
	Baseline FOIS	3.48 (2.31–5.28)	5.94	< 0.001
	Stroke type	2.25 (0.63–8.57)	1.25	0.211
	NIHSS	0.96 (0.87–1.06)	−0.75	0.451
	mRS	0.91 (0.40–2.09)	−0.21	0.831
	Duration	1.02 (0.94–1.10)	0.49	0.623

OR, odds ratio; CI, confidence interval.

For FOIS, an OR > 1 indicates a higher probability of achieving a better (higher) functional level.

For PAS, an OR < 1 indicates a lower probability of being in a worse (higher) penetration-aspiration category.

The model was adjusted for all variables listed in the table.

In the propensity score-adjusted sensitivity analysis, the direction of the estimates for the primary and key secondary outcomes was generally consistent with the main adjusted analyses. The association between the intervention and lower post-treatment PAS score remained statistically significant after additional adjustment for the propensity score. Details are provided in Supplementary Table 3.

### Safety and adverse events

Adverse events were identified from routine treatment records and nursing/rehabilitation notes rather than collected using a prespecified standardized adverse-event form. No serious adverse events were reported in either group during the 3-week treatment period. Mild fatigue during tongue pressure resistance training was occasionally observed and resolved after rest; however, the exact frequency was not systematically recorded. No mucosal injury, electrical burn, or treatment discontinuation due to intolerance was reported. Because adverse events were not collected using a standardized checklist, safety findings should be interpreted cautiously.

## Discussion

PSD is a multifactorial neurological deficit characterized by impaired supratentorial or brainstem regulation over the complex sensorimotor integration required for deglutition. The resultant muscle weakness, delayed swallowing reflex, and inadequate laryngeal elevation are the primary pathological drivers of aspiration and subsequent pneumonia (20). While conventional rehabilitation focuses on basic compensatory techniques and static electrical stimulation, its clinical improvement is often constrained by a lack of physiological specificity. The present study aimed to investigate short-term swallowing outcomes associated with a combined rehabilitation strategy comprising VFS-guided inductive NMES and active tongue pressure resistance feedback training for PSD. The clinical relevance of the observed effect sizes should be interpreted cautiously. Although the adjusted between-group difference in PAS score was statistically significant, the magnitude of the adjusted difference was modest and smaller than one full PAS category. Therefore, this finding should not be interpreted as demonstrating a large individual-level reduction in aspiration severity. Rather, the overall pattern of results, including improvements in FOIS, SWAL-QOL, tongue pressure, hyolaryngeal displacement, and swallowing time, suggests a consistent short-term association between the integrated intervention and swallowing-related recovery. Because universally accepted minimal clinically important differences for PAS, FOIS, and several VFS kinematic parameters in PSD are not well established, future studies should incorporate predefined clinically meaningful thresholds and patient-centered outcomes. A possible explanation for the observed associations may involve enhanced sensory-motor input and task-specific swallowing practice. First, regarding inductive neuromuscular electrical stimulation, traditional static NMES predominantly applies current to the skin overlying the anterior neck, which struggles to sufficiently activate deep pharyngeal or intraoral musculature. Inductive mobile stimulation utilizes a handheld probe, allowing therapists to directly target a wider array of key anatomical structures, including the intraoral tongue, soft palate, uvula, and posterior pharyngeal wall. This localized electrical stimulation may provide additional sensory-motor input and may modulate peripheral neuromuscular excitability. By providing intense, dynamic, and localized sensory input, this technique may theoretically facilitate swallowing-related motor practice and sensory feedback, although neural reorganization was not directly assessed in this study (21). Our sEMG results showed a shorter swallowing duration in the intervention group after adjustment, whereas the adjusted between-group difference in maximum sEMG amplitude did not reach conventional statistical significance. Therefore, the sEMG findings should be interpreted as suggesting improved swallowing-related muscle activity rather than definitive evidence of enhanced neuromuscular recruitment.

The integration of tongue-pressure resistance feedback training targets a critical component of bolus propulsion and swallowing biomechanics. The tongue is not merely an oral manipulator, and its robust contact with the hard palate generates the pressure required for posterior bolus propulsion propelling the bolus posteriorly into the pharynx. Furthermore, the base of the tongue anatomically anchors the suprahyoid muscle group. According to Wang and Tong (12), who investigated tongue pressure biofeedback in head and neck tumor patients, visual feedback systems may improve patient engagement and training adherence. Traditional therapy often leaves the patient guessing the magnitude of their effort. Our biofeedback system visualizes isometric tongue pressure in real-time, functioning as a real-time visual feedback tool. It transforms a passive, repetitive exercise into a targeted, goal-oriented cognitive-motor task. By setting dynamic goals at 60%–80% of the maximum voluntary capacity, the training may help improve tongue pressure generation through repeated resistance practice. Yeates et al. (22) also reported that tongue-pressure training may improve tongue strength and pressure-generation precision in individuals with dysphagia. Our VFS kinematic data are consistent with these findings, demonstrating increased superior and anterior excursion of both the hyoid bone and the thyroid cartilage. Such improved hyolaryngeal elevation is thought to enhance airway protection via epiglottic closure and helps generate negative pressure for upper esophageal sphincter opening, which may reduce the risk of pyriform sinus residue and aspiration.

The guidance of VFS facilitates personalized targeted treatment planning for these interventions. Dysphagia is highly heterogeneous; a patient with predominant oral preparatory deficits requires entirely different parameters than a patient with a delayed pharyngeal trigger. By utilizing baseline VFS, our therapists were able to accurately identify whether the anatomical level of impairment involved insufficient base of tongue retraction, delayed hyoid elevation, or unilateral pharyngeal weakness. This allowed the mobile electrical stimulation to be applied precisely to the paralyzed or weakened segments, realizing an individualized assessment-guided treatment approach.

Clinically, these physiological improvements carry significant potential implications for patient recovery. The increase in FOIS scores suggests improvement in functional oral intake during the short-term treatment period, although longer follow-up is required to determine whether these gains translate into sustained dietary independence and reduced aspiration-related complications. Accompanying this functional recovery is a marked improvement in the SWAL-QOL scores. As dysphagia frequently triggers anxiety, depression, and social isolation, the improvement in SWAL-QOL suggests a reduced dysphagia-related quality-of-life burden, although anxiety, depression, and social participation were not directly measured in the present study (12).

### Limitations and future directions

Several limitations should be acknowledged. First, this was a single-center, non-randomized prospective study with patient preference-based allocation. Although adjusted analyses were performed to account for measured baseline imbalances, residual confounding from unmeasured factors such as rehabilitation motivation, family support, socioeconomic status, and adherence cannot be excluded. Therefore, the findings should be interpreted as associations rather than definitive causal evidence. Second, although the total therapist-supervised treatment duration was matched between groups, the intervention was multicomponent. Inductive electrical stimulation, tongue pressure resistance feedback training, and VFS-guided individualized treatment planning were delivered as an integrated program, making it impossible to determine the independent effect of each component. Future factorial, sham-controlled, or component-specific randomized studies are needed. Third, the follow-up period was limited to 3 weeks. Longer follow-up is required to determine whether the observed short-term improvements persist and whether they reduce clinically important outcomes such as aspiration pneumonia, tube-feeding dependence, malnutrition, and long-term disability. Fourth, although outcome assessments were performed by independent rehabilitation physicians blinded to treatment allocation, patients and treating therapists could not be blinded because of the nature of the intervention. Therefore, performance bias cannot be fully excluded. In addition, formal inter-rater reliability testing for VFS-derived outcomes was not performed because duplicate independent ratings were not prospectively recorded. Measurement variability in PAS grading and kinematic parameters therefore cannot be fully excluded. Fluoroscopy time and radiation dose were not systematically recorded in the research database. Fifth, maximum sEMG amplitude was reported as raw peak amplitude without normalization to maximum voluntary isometric contraction. Raw sEMG amplitude is sensitive to electrode placement, subcutaneous tissue thickness, skin preparation, and skin impedance. Therefore, sEMG amplitude findings should be interpreted as exploratory indicators of submental muscle activity rather than direct measures of muscle strength or neuromuscular recruitment. Finally, no formal *a priori* sample-size calculation was performed, and multiple secondary outcomes were analyzed without adjustment for multiplicity. Therefore, secondary outcome findings should be interpreted as exploratory. Larger multicenter randomized controlled trials with predefined primary endpoints, formal sample-size estimation, blinded assessment, standardized adverse-event monitoring, and longer follow-up are warranted to validate these findings. In addition, this study was not prospectively registered in a public trial registry, and therefore prespecification of outcomes and analyses cannot be independently verified. No randomization, matching, or balancing procedure was used for group allocation. Formal testing of proportional odds assumptions was not performed, which is acknowledged as a limitation. Adverse events were extracted from routine clinical records rather than collected using a prespecified standardized checklist. Therefore, minor adverse events may have been underreported, and safety findings should be interpreted cautiously.

## Conclusion

In this preference-based, non-randomized prospective study, VFS-guided inductive electrical stimulation combined with tongue pressure resistance feedback training was associated with greater short-term improvements in PAS score, functional oral intake, swallowing-related quality of life, tongue pressure, hyolaryngeal kinematics, and swallowing time compared with conventional rehabilitation. However, because treatment allocation was not randomized and the intervention was multicomponent in nature, the findings should be interpreted as associations rather than definitive causal evidence. Larger randomized, time-matched, multicenter studies with longer follow-up are warranted to validate these results.

## Data Availability

The raw data supporting the conclusions of this article will be made available by the authors, without undue reservation.
