# Supplementary material for: Short-term swallowing outcomes associated with inductive electrical stimulation combined with tongue pressure resistance feedback training in post-stroke dysphagia
Source: Front Med (Lausanne). 2026 Sep 1;13:1911750. doi: 10.3389/fmed.2026.1911750 (PMC13574561; doi:10.3389/fmed.2026.1911750)
Supplement: Supplementary file 1 [file Table1.docx]

Supplementary Table 1. Component-level summary of treatment content and duration

| Component | Control group | Intervention group |
| --- | --- | --- |
| Total supervised duration | 30 min/session | 30 min/session |
| Frequency | Once daily, 5 days/week, 3 weeks | Once daily, 5 days/week, 3 weeks |
| Oral sensory-motor training | Included | Incorporated as clinically indicated |
| External NMES | Included | Modified/replaced by mobile inductive stimulation when appropriate |
| Swallowing maneuvers | Included | Included/combined with stimulation |
| Direct feeding training | VFS-guided | VFS-guided |
| Inductive electrical stimulation | Not performed | Approximately 20 min within same 30-min session |
| Intraoral stimulation sites | Not applicable | Tongue, soft palate, palatopharyngeal arch, uvula, posterior pharyngeal wall |
| Tongue pressure feedback training | Not performed | Incorporated within same 30-min session |
| Therapist contact time | 30 min | 30 min |
