# Supplementary material for: Short-term swallowing outcomes associated with inductive electrical stimulation combined with tongue pressure resistance feedback training in post-stroke dysphagia
Source: Front Med (Lausanne). 2026 Sep 1;13:1911750. doi: 10.3389/fmed.2026.1911750 (PMC13574561; doi:10.3389/fmed.2026.1911750)
Supplement: Supplementary file 2 [file Table2.docx]

Supplementary Table 2：Treatment adherence and intervention fidelity

| Variable | Control group | Intervention group |
| --- | --- | --- |
| Planned supervised sessions per patient | 15 | 15 |
| Total planned supervised sessions | 1785 | 1785 |
| Completed supervised sessions | 1785 | 1785 |
| Missed sessions | 0 | 0 |
| Interrupted sessions | 0 | 0 |
| Treatment discontinuation | 0 | 0 |
| Mean session duration | 30 min | 30 min |
| Therapist-supervised duration matched | Yes | Yes |
