# Supplementary material for: Short-term swallowing outcomes associated with inductive electrical stimulation combined with tongue pressure resistance feedback training in post-stroke dysphagia
Source: Front Med (Lausanne). 2026 Sep 1;13:1911750. doi: 10.3389/fmed.2026.1911750 (PMC13574561; doi:10.3389/fmed.2026.1911750)
Supplement: Supplementary file 3 [file Table3.docx]

Supplementary Table 3. Propensity score-adjusted sensitivity analyses for primary and key secondary outcomes

| Outcome | Effect measure | Main adjusted estimate (95% CI) | *P* value | PS-adjusted estimate (95% CI) | *P* value |
| --- | --- | --- | --- | --- | --- |
| PAS score | Mean difference | -0.21 (-0.37 to -0.05) | 0.012 | -0.48 (-0.65 to -0.31) | <0.001 |
| FOIS score | Mean difference | 0.35 (0.19 to 0.52) | <0.001 | 0.56 (0.38 to 0.74) | <0.001 |
| SWAL-QOL | Mean difference | 14.30 (9.49 to 19.20) | <0.001 | 19.85 (14.20 to 25.50) | <0.001 |
| SSA score | Mean difference | -0.70 (-1.18 to -0.21) | 0.005 | -1.02 (-1.45 to -0.59) | <0.001 |
| Tongue pressure | Mean difference | 5.65 (5.06 to 6.25) | <0.001 | 8.92 (7.45 to 10.39) | <0.001 |
| Swallowing time | Mean difference | -0.04 (-0.07 to -0.01) | 0.013 | -0.07 (-0.11 to -0.03) | <0.001 |
| Raw sEMG amplitude | Mean difference | 10.70 (-0.05 to 21.40) | 0.051 | 14.25 (2.80 to 25.70) | 0.015 |
| Favorable status | Odds Ratio (OR) | 1.71 (1.15 to 2.59) | 0.009 | 2.34 (1.52 to 3.60) | <0.001 |

Main adjusted estimates were derived from ANCOVA models (for continuous outcomes) or logistic regression (for favorable status), adjusted for baseline PAS score, stroke type, NIHSS score, modified Rankin Scale (mRS) score, and disease duration. PS-adjusted estimates were derived from models further adjusted for the propensity score as a covariate. The propensity score was calculated using a logistic regression model including stroke type, mRS, lesion location, baseline FOIS, baseline SSA, baseline SWAL-QOL, baseline tongue pressure, pneumonia before intervention, and diabetes mellitus. Favorable swallowing status was defined as a Kubota Water Swallow Test grade of I or II. P<0.05P < 0.05P<0.05 was considered statistically significant. PAS, Penetration-Aspiration Scale; FOIS, Functional Oral Intake Scale; SWAL-QOL, Swallowing Quality of Life; SSA, Standardized Swallowing Assessment; sEMG, surface Electromyography; OR, odds ratio; CI, confidence interval; PS, propensity score.
